# Injury Patterns in Krav Maga Training: A Cross-Sectional Study

**DOI:** 10.7759/cureus.75619

**Published:** 2024-12-12

**Authors:** Eyal Yaacobi, Tal Shachar, Dafna Shilo Yaacobi, Omer Marom, Shanny Gur, Alex Lvovsky, Shlomi Abeceedon, Nissim Ohana

**Affiliations:** 1 Orthopaedics, Meir Medical Center, Kfar Saba, ISR; 2 Orthopaedic Surgery, Meir Medical Center, Kfar Saba, ISR; 3 Plastic and Reconstructive Surgery, Rabin Medical Center, Petach Tikva, ISR; 4 Dentistry, Goverment Services, Kfar Saba, ISR; 5 Sports Medicine, Wingate Institute for Physical Education and Sports, Netanya, ISR

**Keywords:** athletes, krav-maga, martial arts injuries, non-competitive sport, sport injury

## Abstract

This cross-sectional study, conducted from July 2023 to January 2024, examines injury patterns and associated risk factors among Krav Maga athletes to develop tailored prevention strategies for this unique martial art. A survey of 109 participants revealed that 65 injuries were reported, with 59.6% caused by actions from another individual and 24.8% (27 cases) being self-inflicted. The most frequently injured areas were the lower limbs (42 participants, 38.5%), torso (19 participants, 17.4%), and upper limbs (19 participants, 17.4%). Sprains or ligament tears were the most common diagnosis, accounting for 35 cases (32.1%), followed by fractures in 24 cases (22%). Participants ranged in age from 10 to 65 years, with males (95 participants, 87.2%) experiencing more injuries than females. Significant disparities were noted in injury rates based on training intensity (p < 0.05). Recovery periods varied, with 21 participants (19.3%) reporting durations exceeding 30 days. These findings underscore specific injury patterns and risk factors in Krav Maga, highlighting the importance of tailored prevention strategies focusing on flexibility, strength, and proper techniques to reduce injury risks.

## Introduction

Krav Maga and combat sports differ fundamentally in their objectives, techniques, and training methodologies [1,2]. Krav Maga, developed for the Israel Defense Forces, is a military self-defense system designed to neutralize threats in real-world scenarios, unlike competitive martial arts such as karate or taekwondo [2-5]. It prepares individuals to confront and survive violent encounters [6,7]. Krav Maga training includes a variety of techniques, such as strikes, grapples, and the use of improvised weapons, with scenarios reflecting the unpredictability of real-world violence [8]. As an intense hand-to-hand combat system, practitioners are exposed to rigorous training scenarios designed to simulate stressful and potentially harmful encounters [7].

Civilian Krav Maga has gained increasing popularity worldwide as a means of self-defense, fitness, and personal empowerment, with estimated global participation involving thousands of practitioners and instructors, spanning gyms, martial arts schools, and fitness centers globally [8]. However, this growing civilian application raises questions about its safety, particularly regarding the risks and patterns of injury among non-military practitioners. Unlike competitive martial arts, civilian Krav Maga training emphasizes practical survival skills rather than standardized competition, resulting in unique training demands and injury risks.

Despite its unique methodology, injury trends in Krav Maga may parallel those observed in other martial arts and combat sports. Studies on similar disciplines, such as judo, karate, and taekwondo, report common injuries, including sprains, contusions, and fractures, often influenced by training intensity, participant skill levels, and the types of maneuvers practiced [9-11]. The injury patterns in Krav Maga, while underexplored, likely share these influences and are particularly relevant for instructors, practitioners, and healthcare professionals who aim to ensure safety without compromising effectiveness.

The aim of our study is to characterize the injury patterns of Krav Maga athletes training in approved facilities and to identify the risk factors associated with these injuries. By doing so, we aim to establish guidelines for primary prevention and improve the safety of Krav Maga training.

This study seeks to address the following specific research questions: (1) What are the most common injury patterns observed among Krav Maga practitioners? (2) How do demographic and training-related factors influence the risk and severity of injuries in Krav Maga? (3) What preventive strategies can be derived from understanding these injury patterns to improve safety in Krav Maga training?

## Materials and methods

This cross-sectional study was conducted from July 2023 to January 2024 and recruited athletes practicing Krav Maga Israel (KMI) at gyms affiliated with the Orthopedic Department of Meir Medical Center in Kfar Saba, Israel. The study protocol was approved by the internal review board (approval number 0071-22-MMC) and conducted in adherence to the principles of the Declaration of Helsinki. Completion of the questionnaire indicated informed consent, as detailed in the participant information sheet approved by the ethics committee.

Study population

The study population included athletes training at a single officially registered non-combat sports facility that receives medical support from our department. Eligibility criteria included active participation in Krav Maga training and completion of the questionnaire. Participants were excluded if they provided incomplete data or reported an existing injury at the time of the study. Informed consent forms and the research team's contact details were attached to all distributed questionnaires. Withdrawal from the study was permitted at any stage without consequence.

​​​​​​Sample size calculation and recruitment

The sample size was determined based on an estimated injury prevalence of 30%, requiring at least 100 participants to achieve 80% statistical power with a 5% significance level. A total of 149 questionnaires were distributed, yielding 109 complete responses. Participants were recruited over a three-month period, from July 2023 to January 2024.

Selection Criteria 

Only trainees practicing at the index facility under the supervision of registered instructors were included in the study to ensure consistent training standards and adherence to professional guidelines.

​​​​​​*Questionnaire Validation*

The questionnaire was adapted and validated based on prior studies examining martial arts injuries [9,11]. Validation involved expert review by sports medicine professionals and a pilot study with 20 participants.

Definitions and Classifications

Definition of 'Severe Injury': Severe injuries were defined as those requiring surgical intervention, prolonged recovery exceeding 30 days, or causing functional limitations. Classification of Injury Types: Injuries were categorized into sprains, fractures, soft tissue trauma, and other types based on treatment needs and recovery times. Training Intensity Categorization: Training intensity was categorized as minimal, moderate, or rigorous based on self-reported weekly training hours and perceived physical effort during sessions.

Data Collection

The survey collected comprehensive data on (a) demographics: age, sex, height, and weight; (b) lifestyle factors: daily activities, dietary habits, smoking status, and sleep duration; (c) training-related variables: number of injuries during training, causes and types of injuries, injury locations, treatment methods, and recovery duration.

Statistical Analysis

Data were summarized as mean ± standard deviation (SD) for continuous variables and as frequencies and percentages for categorical variables. Univariate analysis was conducted using Chi-square or Fisher’s exact tests for categorical data, and one-way ANOVA for continuous variables. Post-hoc Bonferroni comparisons were applied to obtain significant ANOVA results. Statistical significance was set at p < 0.05. All analyses were performed using R version 3.6.1 (http://www.R-project.org/).

Justification for statistical tests: Chi-square and Fisher’s exact tests were used to analyze categorical variables to handle small sample sizes, while one-way ANOVA was used for continuous variables to compare means across groups. Treatment of missing data: incomplete responses were excluded from analysis if critical variables were missing. Non-critical missing data were handled using mean imputation for continuous variables and mode imputation for categorical variables. Subgroup analysis criteria: subgroup analyses were conducted based on age, sex, and training intensity to identify demographic and training-related factors associated with injury patterns.

## Results

Out of 149 distributed questionnaires, 109 completed responses were received in September 2023, representing a response rate of 73.2%. Table 1 provides a detailed overview of the participants' demographic characteristics, including age, training experience, and prior martial arts experience. Participants ranged in age from 13 to 70 years, with a mean age of 32.4 ± 8.5 years. Training experience ranged from 0.5 to 10 years, with an average of 3.2 ± 1.7 years. Approximately 45% of participants reported prior martial arts experience. Participants were categorized into five age groups: <13 years (five participants, 4.6%), 14-17 years (20 participants, 18.3%), 18-39 years (54 participants, 49.5%), 40-64 years (25 participants, 22.9%), and >65 years (five participants, 4.6%). The majority of respondents were male (95 participants, 87.2%), with females representing 14 participants (12.8%). The average height and weight were 174 cm (SD ± 9.7) and 78 kg (SD ± 20.5), respectively.

**Table 1:** Demographics of participants NA: Not available

Category	Range	Mean ± SD
Age (years)	13–70	32.4 ± 8.5
Training experience (years)	0.5–10	3.2 ± 1.7
Previous martial arts experience (%)	NA	45%

Regarding daily activity levels, 50 participants (45.9%) reported moderate activity, 33 participants (30.3%) reported rigorous activity, and 26 participants (23.9%) reported minimal activity. Among participants, 16 individuals (14.7%) were active smokers, and 103 individuals (94.5%) followed a nonrestrictive diet. A small proportion reported being vegetarians (five participants, 4.6%) or adhering to a gluten-free diet for non-medical reasons (one participant, 0.9%).

Approximately 23 participants (21.1%) reported experiencing one injury during training, 23 participants (21.1%) reported two injuries, and 23 participants (21.1%) reported five or more injuries. Notably, 18 participants (16.5%) reported no injuries. A total of 65 injuries were reported, with 59.6% caused by actions from another individual (e.g., opponent strikes) and 24.8% (27 cases) being self-inflicted. The most common injury mechanisms were leg kicks and improper landings or joint rotations, each accounting for 21 injuries (19.3%).

**Figure 1:**
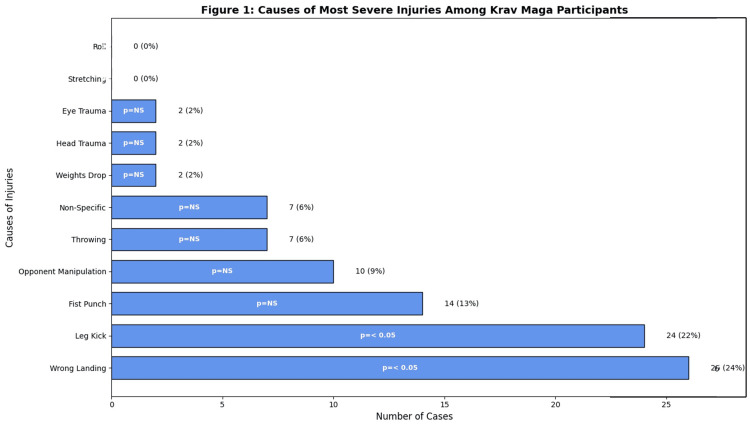
Causes of most severe injuries among Krav Maga participants This figure illustrates the distribution of severe injuries based on their causes among Krav Maga participants. Percentages were calculated as the proportion of injuries for each category relative to the total reported injuries (n = 109).  The most common causes of severe injuries were "Wrong Landing" (24%) and "Leg Kick" (22%), followed by "Fist Punch" (13%) and "Opponent Manipulation" (9%). Statistical significance (p-values) for each injury cause is annotated within the bars, highlighting key differences. For instance, significant differences (p < 0.05) are observed for the top two causes.

Injury rates were calculated per 100 training hours, revealing higher rates among high-intensity trainees compared to those engaged in moderate or minimal-intensity training (p < 0.05). Acute injuries accounted for 78% of cases, while chronic injuries comprised 22%. Approximately 23 participants (21.1%) reported experiencing one injury during training, 23 participants (21.1%) reported two injuries, and 23 participants (21.1%) reported five or more injuries. Notably, 18 participants (16.5%) reported no injuries. The causes of the most severe injuries are shown in Figure 1.

**Figure 2:**
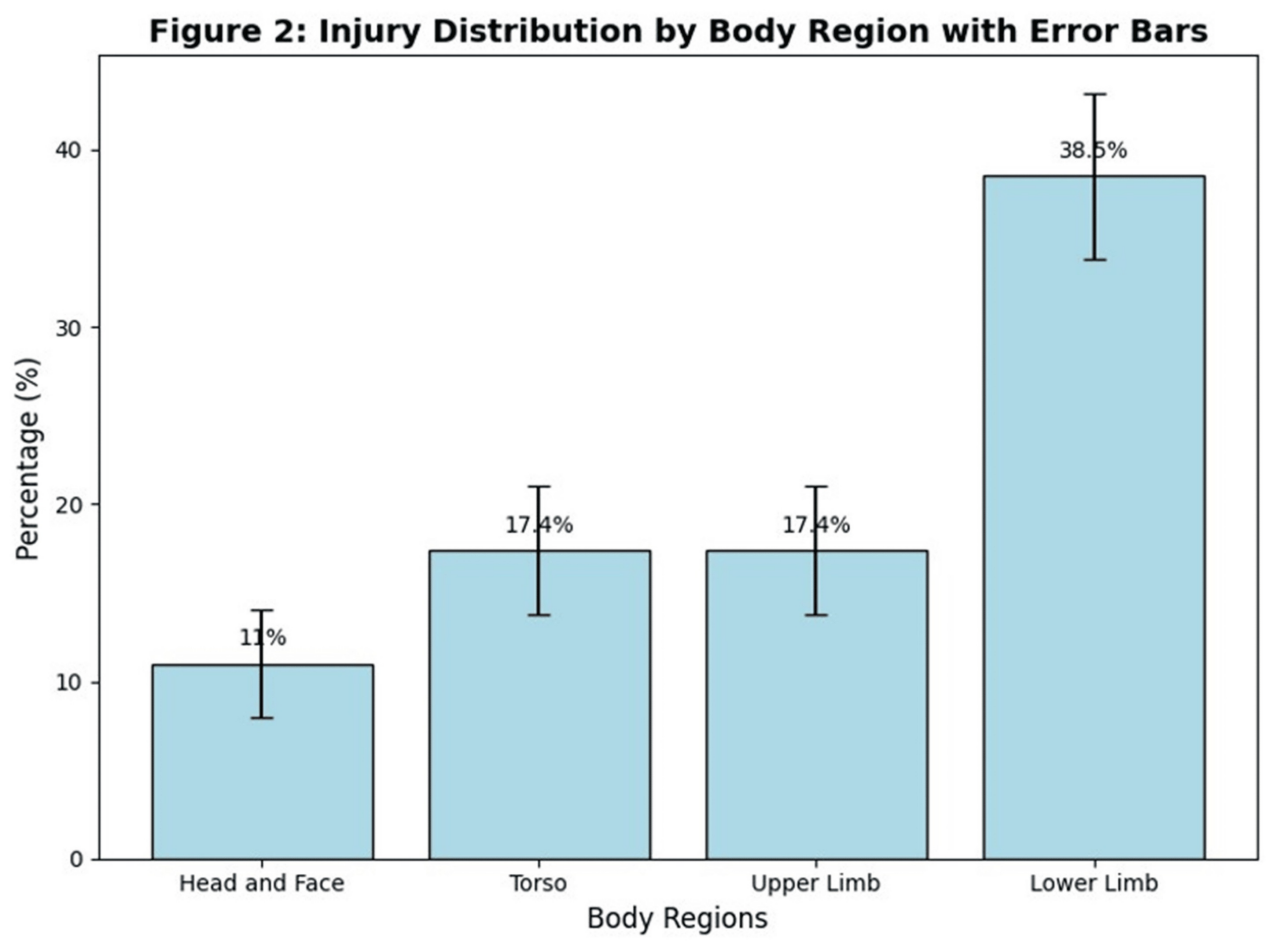
Injury distribution by body region with error bars This figure illustrates the distribution of injuries across different body regions among Krav Maga athletes. The proportions of injuries are represented as percentages, with the most frequently affected region being the lower limbs (38.5%), followed by the torso (17.4%) and upper limbs (17.4%). Head and face injuries were the least common (11%). Error bars indicate ±1 standard error of the proportion, calculated based on the sample size (n = 109).

The distribution of injuries across body regions is illustrated in Figure 2. Lower limb injuries were the most common, affecting 42 participants (38.5%). Injuries to the torso and upper limbs each affected 19 participants (17.4%), while head and face injuries accounted for 12 cases (11%).

**Figure 3:**
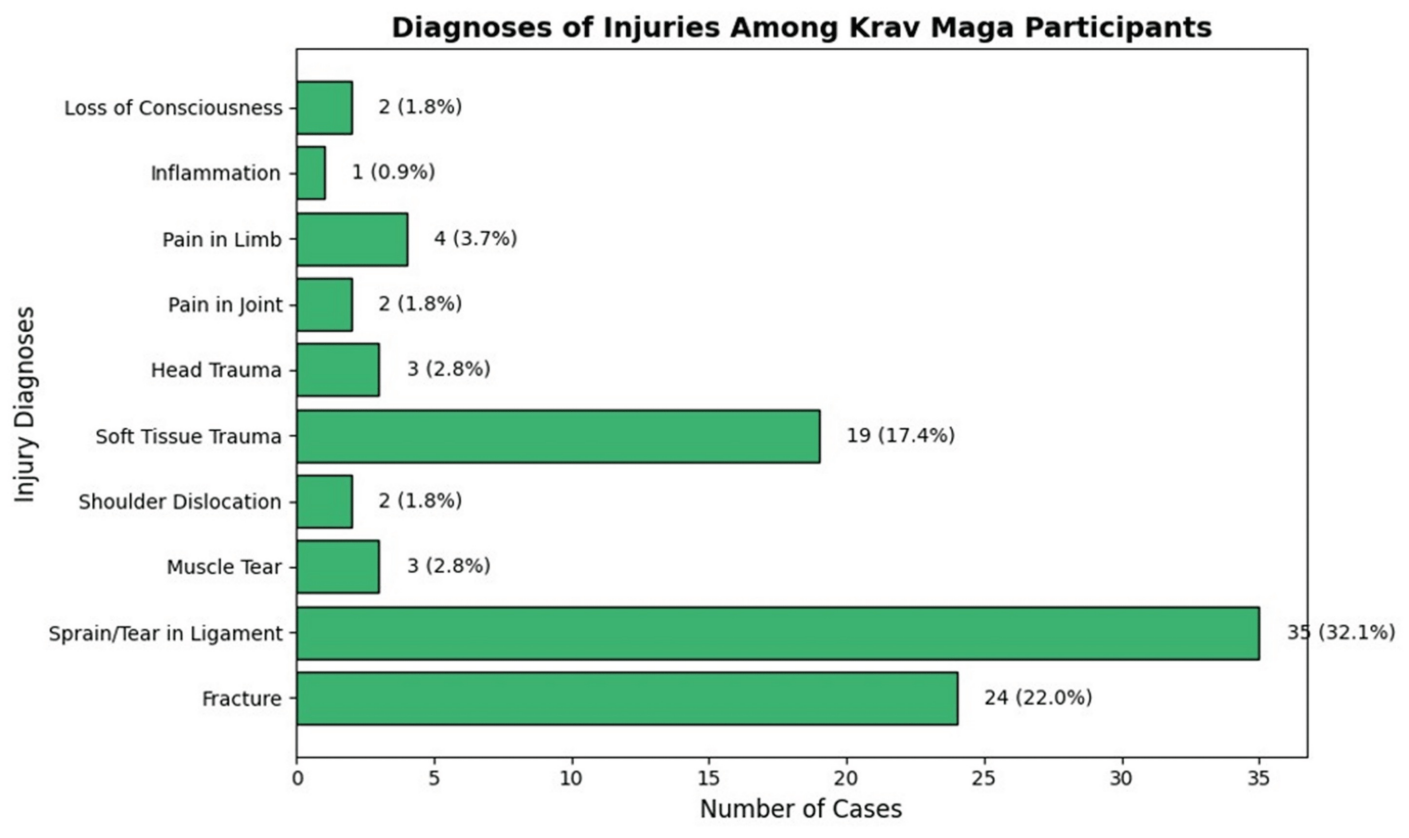
Diagnoses of injuries among Krav Maga participants This figure illustrates the distribution of injury diagnoses among Krav Maga participants. Percentages represent the proportion of injuries for each category relative to the total number of reported injuries (n = 109). The most common injuries were sprains or ligament tears (32.1%), followed by fractures (22%), and soft tissue trauma (17.4%).

Sprains or ligament tears were the most frequently diagnosed injuries, comprising 35 cases (32.1%) (Figure 3). A small proportion of participants, five individuals (4.6%), required surgical intervention, while 36 participants (33.3%) were treated non-operatively with rest and physical therapy. An additional 27 participants (24.8%) required no treatment, and 28 participants (25.7%) received treatment but resumed training immediately.

**Figure 4:**
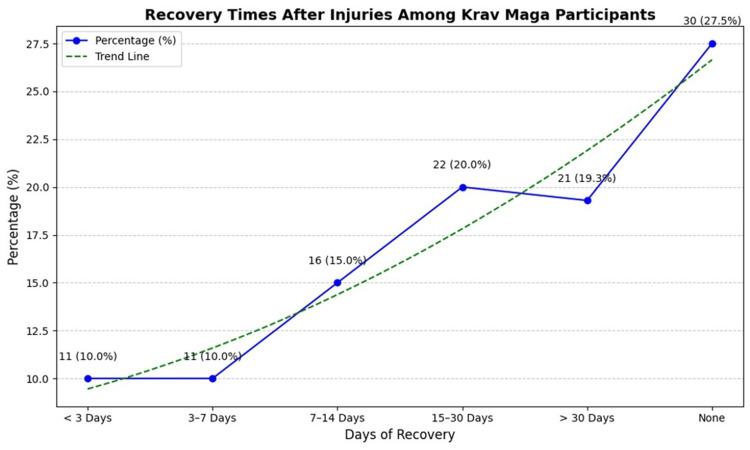
Recovery times after injuries among Krav Maga participants This figure illustrates the distribution of recovery times among injured participants. Percentages were calculated as the proportion of participants in each recovery time category relative to the total injured participants (n = 109). The trend line represents the general pattern of recovery times across categories.

Recovery times varied significantly among participants. Despite the severity of their injuries, 30 participants (27.5%) continued training without interruption. Conversely, 21 participants (19.3%) reported recovery periods exceeding 30 days, during which they refrained from all training activities. These findings underscore the variability in injury severity and highlight the importance of individualized rehabilitation strategies to facilitate recovery and prevent reinjury (Figure 4).

## Discussion

This study identified torso (19 participants, 17.4%) and lower limb injuries (42 participants, 38.5%) as the most frequently injured regions in Krav Maga training, with sprains and ligament tears being the predominant diagnoses (35 cases, 32.1%). Male athletes and those engaging in high-intensity training were at a higher risk of injury.

Key findings

The most common injuries in Krav Maga were sprains and ligament tears, predominantly in the lower limbs and torso. This pattern is consistent with Krav Maga's emphasis on kicking and defensive maneuvers, which place significant strain on joints and ligaments.

Comparison with other martial arts

This contrasts with prior research on martial arts, which often identifies upper extremity injuries, specifically the shoulders, hands, and fingers, as the most common ones. For example, Farkash et al. reported that among military Krav Maga practitioners, upper limb injuries accounted for 31% of cases, predominantly affecting the hands and wrists [8]. This difference likely reflects variations in the training context, with military programs emphasizing intensive striking maneuvers often performed without protective gloves, placing greater stress on the hands. In civilian training, lower limb injuries are more prominent due to the focus on dynamic kicking and footwork techniques.

Additionally, Possley et al. reported that in Modern Army Combative training among U.S. soldiers, the knee, shoulder, and lumbar spine were the most frequently injured body parts [12]. These findings emphasize the variability in injury patterns across martial arts. Compared to taekwondo and judo, Krav Maga demonstrated fewer shoulder injuries but a higher prevalence of lower limb injuries [13].

Interestingly, head and face injuries, frequently reported in other martial arts and combat sports [3-6], were relatively rare in this study (12 participants, 11%). The use of protective measures, such as headgear in civilian Krav Maga training, likely contributed to this lower incidence. This highlights the value of safety equipment in mitigating severe injuries like concussions, which are prevalent in unprotected disciplines like boxing [3,14,15].

Fractures were the second most common injury observed (24 cases, 22%), aligning with findings from other martial arts research [9,16,17]. Notably, none of these fractures required surgical intervention, as all were managed conservatively without complications such as non-union. Repetitive striking, particularly with open or closed palms, is a likely contributor. Farkash et al. similarly identified fractures of the radial ray in military Krav Maga trainees, emphasizing the need for improved protective equipment and refined striking techniques to reduce hand and wrist injuries [8].

Contrary to expectations, shoulder injuries-commonly reported in grappling martial arts [18,19]-were rare in our study (0.9%). This reflects Krav Maga’s defensive and disengagement-oriented training style, which avoids prolonged grappling. Additionally, adherence to safety protocols and proper technique in registered training facilities likely mitigates shoulder injury risks.

Sprains and ligament tears emerged as the most frequently reported diagnoses in our study, consistent with patterns seen in other contact sports [20-22]. The predominance of lower limb injuries likely reflects the physical demands of Krav Maga, which emphasize rapid pivots, defensive kicks, and dynamic footwork [23,24]. These movements place significant stress on joints and ligaments [14,16,25], highlighting the need for injury prevention strategies that focus on improving flexibility, strength, and landing techniques.

Our data suggest that injury frequency correlates with training intensity, particularly among athletes aged 18-39. This age group is more likely to participate in both civilian and military Krav Maga training due to Israel’s mandatory military draft, which begins at age 18. Moreover, the higher incidence of injuries among male athletes likely reflects their greater participation in high-intensity training or more physically demanding sessions. These findings underline the importance of targeted interventions, such as tailored strength training and gender-specific injury prevention programs, to reduce injury risk across diverse practitioner demographics.

Clinical implications

To reduce injury risks, we recommend structured warm-up routines, strengthening exercises for the lower limbs, and the use of protective gear, such as shin guards and headgear. Supervised training sessions focusing on proper technique can further mitigate risks associated with high-intensity training. Incorporating graded intensity training and limiting exposure to high-risk maneuvers, especially for beginners, could also decrease injury incidence. Training programs should consider individual risk factors, such as age, sex, and fitness level.

Limitations

This study has several limitations. The cross-sectional design prevents causal inferences, and self-reported data may introduce recall bias, as participants might not accurately recall all injuries. To mitigate this, future research should consider prospective data collection, such as real-time injury tracking during training sessions.

Additionally, the study population was drawn exclusively from officially registered facilities, potentially limiting generalizability to informal or competitive training settings. Informal training environments may have different injury patterns due to variations in training intensity, techniques, or equipment use. Moreover, the relatively small number of female participants limits the strength of gender-based comparisons.

Finally, other potentially significant factors, such as variations in protective equipment use, instructor experience, or individual fitness levels, were not accounted for. Future studies should include these factors to provide a more comprehensive understanding of injury risks in Krav Maga.

Future research directions

Future studies should employ longitudinal designs to track injury trends over time and evaluate the effectiveness of targeted prevention strategies. Research into the role of protective equipment and instructor experience in injury prevention would also be valuable. Additional studies comparing injury patterns across different martial arts could offer deeper insights into unique risk factors and mitigation strategies.

Our findings provide critical insights for medical professionals and trainers, emphasizing the need for tailored injury prevention programs and modifications to Krav Maga training protocols to enhance safety.

## Conclusions

Our study identifies torso and lower limb injuries as the most common in Krav Maga training, with sprains and ligament tears being the predominant diagnoses. Protective measures, such as headgear, reduce severe injuries, highlighting their importance. Tailored injury prevention strategies focusing on improving flexibility, strength, and proper technique could significantly reduce the risk of injuries. Furthermore, understanding the role of training intensity and participant demographics in injury patterns can inform more personalized approaches to injury prevention. Future research should explore long-term outcomes, evaluate the effectiveness of specific protective gear, and assess the impact of different training regimens on injury rates.
